# Right atrial appendage thrombosis after percutaneous left atrial appendage occlusion: a case report

**DOI:** 10.3389/fcvm.2026.1868125

**Published:** 2026-06-17

**Authors:** Haohui Du, Lijun Zeng, Xiaobo Pu

**Affiliations:** 1 Department of Cardiology, Yingshan County People’s Hospital, Nanchong, China; 2 Department of General Internal Medicine, West China Second University Hospital, Sichuan University, Chengdu, China; 3 Department of Cardiology, West China Hospital, Sichuan University, Chengdu, China

**Keywords:** anticoagulation, atrial cardiomyopathy, atrial fibrillation, dilated cardiomyopathy, left atrial appendage occlusion, right atrial appendage, thrombosis

## Abstract

Left atrial appendage occlusion (LAAO) is an established alternative stroke-prevention strategy for patients with atrial fibrillation (AF) who have contraindications to long-term oral anticoagulation. However, LAAO addresses only left-sided thromboembolism and does not eliminate the risk of thrombus formation in other cardiac chambers. We present the case of a 72-year-old man with dilated cardiomyopathy and persistent AF who developed right atrial appendage (RAA) thrombosis one year after percutaneous LAAO with an Amplatzer Cardiac Plug (ACP). The patient had initially undergone LAAO due to intracerebral hemorrhage during oral anticoagulant therapy. He presented to the emergency department with decompensated heart failure. Transthoracic echocardiography revealed a mobile mass (30 mm × 19 mm) in the right atrium, and contrast-enhanced cardiac computed tomography confirmed a filling defect within the RAA, consistent with RAA thrombus. Anticoagulation with rivaroxaban 15 mg once daily was initiated, and follow-up imaging at two weeks demonstrated complete resolution of the mobile thrombus. This case highlights the potential for RAA thrombosis in patients with AF following LAAO, particularly in the presence of heart failure and enlarged cardiac chambers. Clinicians should remain vigilant for right-sided intracardiac thrombi in this population, even after successful LAAO, and the decision to discontinue anticoagulation after LAAO should be carefully individualized.

## Introduction

Atrial fibrillation (AF) is the most common sustained cardiac arrhythmia in adults with prevalence increasing significantly in patients over 80 years of age (1, 2). AF is associated with a five-fold increase in the risk of ischemic stroke, primarily due to thrombus formation in the left atrial appendage (LAA), which accounts for approximately 90% of intracardiac thrombi in patients with nonvalvular AF (3). For patients who cannot tolerate long-term oral anticoagulation, percutaneous left atrial appendage occlusion (LAAO) has emerged as a viable alternative strategy for stroke prevention (4).

However, LAAO exclusively targets the LAA and does not address the risk of thrombus formation in other cardiac chambers, including the right atrial appendage (RAA). Although RAA thrombosis is considerably less common than LAA thrombosis, it is not negligible. Studies employing transesophageal echocardiography (TEE) to guide cardioversion in AF patients have reported the incidence of RAA thrombosis ranging from 0.4% to 0.73%, compared with 6%–18% for LAA thrombosis (5, 6). Notably, RAA thrombi may reach larger dimensions than their left-sided counterparts, and they carry the risk of pulmonary embolism or paradoxical systemic embolization through a patent foramen ovale (7).

A growing number of case reports have described RAA thrombosis occurring after LAAO or surgical LAA exclusion, raising concerns about the residual thrombotic risk in these patients (8–10). The discontinuation of anticoagulation following LAAO may contribute to an increased risk of right-sided thrombus formation, particularly in patients with additional risk factors such as heart failure, atrial enlargement, and reduced appendage flow velocities. Furthermore, emerging evidence suggests that atrial cardiomyopathy—characterized by structural and functional atrial remodeling, fibrosis, and endothelial dysfunction—creates a persistent thrombogenic substrate that extends beyond the LAA (11). Despite the clinical significance of this entity, there is a paucity of data regarding its prevalence, optimal management, and long-term outcomes.

We report a case of a 72-year-old man with dilated cardiomyopathy (DCM) and persistent AF who developed RAA thrombosis one year after percutaneous LAAO with an Amplatzer Cardiac Plug (ACP), successfully treated with rivaroxaban. This case underscores the importance of vigilant monitoring for right-sided thrombi in AF patients post-LAAO and the need for individualized decision-making regarding anticoagulation discontinuation.

## Case presentation

A 72-year-old man with a history of dilated cardiomyopathy and persistent atrial fibrillation presented to the emergency department with worsening dyspnea on exertion and progressive bilateral lower extremity edema over the preceding 10 days, consistent with decompensated heart failure. His CHA_2_DS_2_-VA score was 3 (heart failure, age 65–74 years, vascular disease), and his HAS-BLED score was 3. His medical history included recurrent palpitations and exertional dyspnea for two years, with a prior diagnosis of paroxysmal AF that had progressed to persistent AF. One year earlier, he had undergone percutaneous LAAO with an Amplatzer Cardiac Plug (ACP; St. Jude Medical, St. Paul, MN, USA) at a local hospital due to an episode of intracerebral hemorrhage that occurred during oral anticoagulant therapy.

Following the LAAO procedure, the patient received dabigatran 110 mg twice daily for six months as post-procedural anticoagulation, after which anticoagulation was discontinued and replaced with aspirin. The post-LAAO antithrombotic strategy of single anticoagulant (reduced-dose dabigatran) followed by aspirin monotherapy was chosen based on the patient's high intracranial hemorrhage risk. Dual antiplatelet therapy was specifically avoided due to bleeding concerns. The patient had no history of hypertension, diabetes mellitus, autoimmune diseases, or endocrine disorders. He denied any history of chronic alcohol use or tobacco use.

On physical examination, his vital signs revealed a temperature of 36.6 °C, pulse rate of 75 beats per min (irregular), respiratory rate of 20 breaths per min, and blood pressure of 107/76 mmHg. He appeared chronically ill with jugular venous distension. Cardiac examination revealed an enlarged heart border to the left, irregular rhythm, and a systolic blowing murmur at the mitral valve auscultation area. No peripheral edema was noted at the time of examination. Chest and abdominal examinations were otherwise unremarkable.

Laboratory investigations revealed serum creatinine of 1.1 mg/dL with an estimated creatinine clearance (Cockcroft-Gault) of approximately 52 mL/min. Serum sodium was 128 mmol/L. C-reactive protein and procalcitonin levels were within normal limits.

Electrocardiography (ECG) demonstrated atrial fibrillation with a ventricular rate of 78 beats per min and complete left bundle branch block (LBBB) with a QRS duration of 130 ms. Ambulatory Holter monitoring revealed slow AF with a mean heart rate of 49 beats per min.

**Figure 1:**
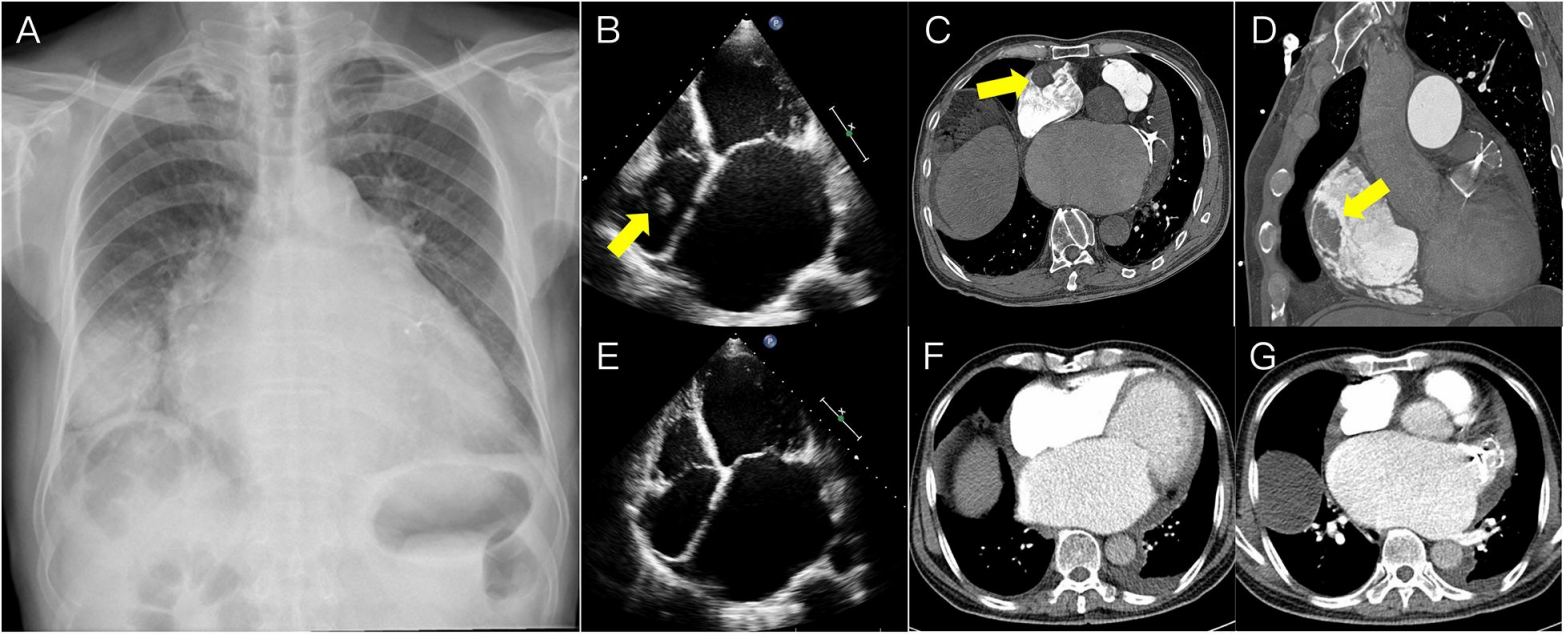
Imaging findings of right atrial appendage thrombosis and its resolution. **(A)** Chest radiograph demonstrating cardiomegaly with the left atrial appendage occluder device visible within the cardiac silhouette. **(B)** Transthoracic echocardiography showing a mobile mass (30 × 19 mm, arrow) in the right atrium. **(C)** and **(D)** Contrast-enhanced cardiac computed tomography confirming a filling defect within the right atrial appendage (arrows). **(E)** Transthoracic echocardiography at two weeks demonstrating complete resolution. **(F)** and **(G)** Contrast-enhanced cardiac CT at two weeks demonstrating resolution.

Chest radiography demonstrated significant cardiomegaly with the LAA occluder device visible within the cardiac silhouette, increased pulmonary vascular markings, bilateral atelectasis more prominent at the right lower lobe attributed to the bilateral pleural effusions (Figure 1A). No clinical evidence of active pulmonary infection was identified.

Transthoracic echocardiography (TTE) revealed severely dilated left atrium (anteroposterior diameter 52 mm) and left ventricle (end-diastolic diameter 72 mm) with severe left ventricular systolic dysfunction [left ventricular ejection fraction (LVEF) 35%]. The right atrium was visually enlarged. Spontaneous echo contrast was present in the left atrium. Additionally, a mobile mass measuring 30 mm × 19 mm was identified in the right atrium, arising from the region of the right atrial appendage (Figure 1B, Supplementary Video S1). Contrast-enhanced cardiac computed tomography (CT) angiography confirmed a filling defect located within the RAA, consistent with RAA thrombus (Figures 1C, D). No device-related thrombus was identified; the Amplatzer Cardiac Plug appeared well-positioned with adequate seal and no significant peri-device leak. Importantly, review of the pre-LAAO echocardiography demonstrated no evidence of RAA thrombus.

Transesophageal echocardiography was not performed during this admission due to the patient's hemodynamic instability at presentation. RAA flow velocities were not formally quantified; however, the clinical picture of low cardiac output and the presence of spontaneous echo contrast were consistent with significantly reduced appendage flow.

Anticoagulation therapy with rivaroxaban 15 mg once daily was initiated, with the reduced dose selected based on a careful risk-benefit assessment considering the patient's prior history of intracerebral hemorrhage, borderline renal function (CrCl ∼ 52 mL/min), advanced age, and heart failure. Guideline-directed medical therapy for heart failure was initiated and up-titrated during this hospitalization, including perindopril, metoprolol succinate, spironolactone, furosemide and tolvaptan. Tolvaptan was prescribed for refractory hyponatremia (serum sodium 128 mmol/L) associated with severe heart failure. SGLT2 inhibitor therapy was not initiated during this admission, and transition from ACE inhibitor to sacubitril/valsartan was planned after hemodynamic stabilization at follow-up. After two weeks of anticoagulation, TTE demonstrated complete disappearance of the mobile RAA mass (Figure 1E). Follow-up contrast-enhanced cardiac CT angiography at two weeks also confirmed resolution of the previously identified RAA thrombus (Figures 1F, G).

**Figure 2:**
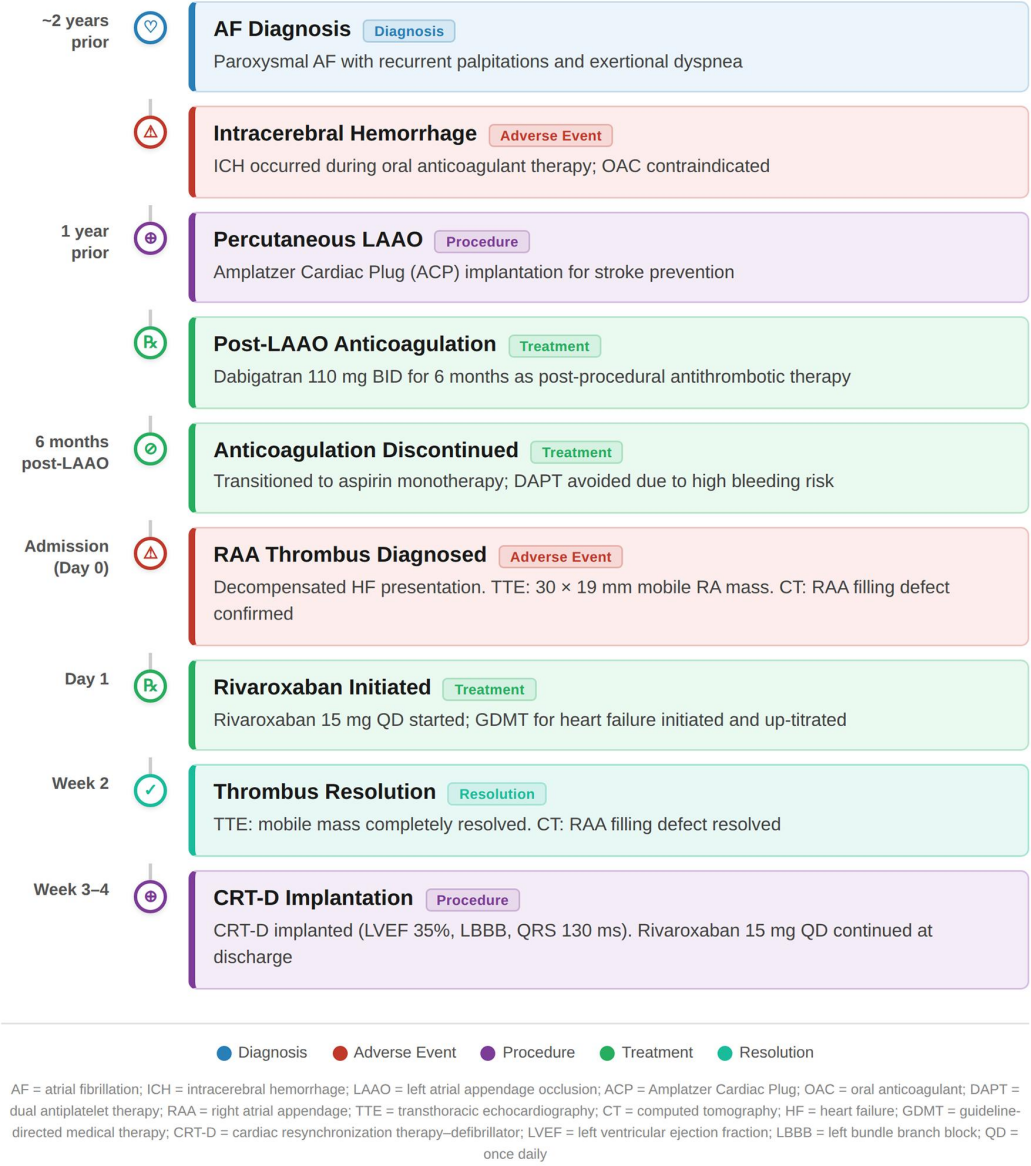
Graphical timeline summarizing the clinical course, including AF diagnosis, intracerebral hemorrhage, LAAO procedure, post-procedural anticoagulation with dabigatran, anticoagulation discontinuation and transition to aspirin, RAA thrombus diagnosis, rivaroxaban initiation, thrombus resolution on TTE and CT, and CRT-D implantation.

Given the patient's severe heart failure with LBBB and LVEF of 35%, he subsequently underwent cardiac resynchronization therapy with defibrillator (CRT-D) implantation (Figure 2). The indication for CRT-D was based on LVEF ≤35%, NYHA class II–III symptoms despite optimized medical therapy, and LBBB morphology with QRS ≥130 ms, representing a Class IIa indication per current guidelines. AV node ablation was discussed but deferred, with reassessment of biventricular pacing percentage planned at follow-up. CRT-D was implanted after the two-week TTE confirmed resolution of the mobile RAA thrombus; the patient remained on rivaroxaban throughout. Post-procedure echocardiography showed improved cardiac function. The patient was maintained on rivaroxaban 15 mg once daily at discharge. At subsequent follow-up, repeat cardiac CT at approximately six weeks showed near-complete resolution of the RAA thrombus with possible minimal residual mural thrombus adherent to the appendage wall, likely reflecting the higher spatial resolution of CT compared to TTE for detecting small non-mobile mural thrombi.

## Discussion

This case illustrates a clinically significant but under-recognized adverse event observed following percutaneous LAAO: the development of RAA thrombosis. While LAAO is increasingly performed as an alternative to long-term anticoagulation for stroke prevention in patients with AF, this case reminds clinicians that occluding the LAA addresses only one potential source of intracardiac thrombus formation.

### Right atrial appendage thrombosis in atrial fibrillation

The pathophysiology of atrial thrombosis in AF is well characterized by Virchow's triad: blood stasis, endothelial dysfunction, and a hypercoagulable state. While the LAA is the predominant site of thrombus formation in nonvalvular AF, the RAA can also serve as a nidus for thrombus development, particularly when similar hemodynamic conditions are present (5, 7). In a landmark TEE study of 983 patients undergoing AF cardioversion, Cresti et al. reported an overall atrial thrombus incidence of 9.7%, with LAA thrombi occurring in 9.3% and RAA thrombi in 0.73% of patients (5). RAA thrombi tended to be larger in size than LAA thrombi. A study of 1,104 patients with persistent AF reported a comparable distribution, with 6% LAA thrombi vs. 0.4% RAA thrombi (6). Autopsy studies have suggested that the true prevalence of right atrial thrombi may be similar to that of left atrial thrombi, raising the possibility that RAA thrombosis is significantly underdiagnosed (7).

Several factors predispose to RAA thrombosis. Reduced RAA flow velocities and the presence of spontaneous echo contrast within the right atrium are independent predictors of RAA thrombus formation (5, 12). Right atrial enlargement and appendage dysfunction also contribute to thrombotic risk (7). The anatomical characteristics of the RAA, which is typically unilobular and triangular shaped with a wider orifice, may partially explain the lower frequency of RAA thrombosis (5, 13). In our patient, several of these risk factors were present, including persistent AF with biatrial enlargement, severe left ventricular dysfunction with secondary right ventricular impairment, and spontaneous echo contrast in the left atrium indicative of a generalized low-flow state.

Emerging evidence supports the concept of atrial cardiomyopathy as a unifying pathophysiological framework for understanding thrombogenesis in AF patients. Atrial cardiomyopathy encompasses structural remodeling, fibrosis, endothelial dysfunction, and impaired contractile function that create a diffuse prothrombotic substrate extending beyond any single appendage (11). This concept is particularly relevant in patients with dilated cardiomyopathy, where biatrial involvement creates conditions favorable for thrombus formation in multiple cardiac chambers, regardless of LAA occlusion status.

### RAA thrombosis after left atrial appendage occlusion

Several case reports and a recent editorial in JACC: Case Reports have drawn attention to the occurrence of RAA thrombosis after LAAO or surgical LAA exclusion (8–10, 13). The mechanism likely involves the discontinuation or reduction of anticoagulation following LAAO, which removes pharmacological protection against thrombus formation in other cardiac chambers. However, the development of RAA thrombosis in this context more likely reflects a multifactorial process involving persistent AF, atrial cardiomyopathy, and advanced structural heart disease, rather than simply anticoagulation withdrawal. In our patient, anticoagulation was discontinued after the initial post-LAAO anticoagulation period, leaving the RAA vulnerable to thrombus formation in the setting of persistent AF, severe left ventricular dysfunction, and biatrial enlargement.

Nakano et al. recently reported a case of RAA thrombosis detected seven months after LAAO in an 88-year-old woman with cardiac amyloidosis and AF (10). Sharma et al. described a patient with a LAAO device found to have RAA thrombus after the post-LAAO anticoagulation period had ended (8). Sakata et al. reported RAA thrombosis two years after thoracoscopic LAA appendectomy, where the thrombus was surgically resected (9). These cases, along with ours, share common features: persistent AF, heart failure, and discontinuation of anticoagulation post-LAAO.

Our case is, to our knowledge, the first reported case of RAA thrombosis following percutaneous LAAO in a patient with dilated cardiomyopathy and severe biatrial enlargement. Prior reports involved cardiac amyloidosis (Nakano et al.) or patients without advanced structural heart disease. Additionally, the rapid and complete resolution of the mobile thrombus within two weeks using reduced-dose rivaroxaban (15 mg daily) represents a distinctive therapeutic observation, as previous cases either used full-dose anticoagulation or required surgical thrombectomy.

In an accompanying editorial, García-Fernández and Cresti emphasized the importance of routine RAA evaluation during TEE examinations in AF patients (13). They recommended the midesophageal bicaval view at 90° with rotation to 130° as the standard approach for RAA visualization.

### Management considerations

The optimal management of RAA thrombus remains unclear due to limited evidence. Treatment options include anticoagulation, thrombolysis, and surgical thrombectomy (8). In our case, rivaroxaban 15 mg once daily achieved complete mobile thrombus resolution within two weeks, consistent with prior reports demonstrating the efficacy of direct oral anticoagulants (DOACs) in resolving intracardiac thrombi (14, 15).

The choice of anticoagulant and dosage warrants careful consideration. Our patient received rivaroxaban 15 mg once daily rather than the standard 20 mg dose, reflecting a balance between effective anticoagulation and the risk of recurrent intracranial hemorrhage. While the patient's estimated CrCl (∼52 mL/min) was slightly above the threshold for dose reduction based on renal function alone, the reduced dose was selected through individualized risk-benefit assessment incorporating prior intracranial hemorrhage, advanced age, and heart failure. This approach is supported by reports demonstrating successful thrombus resolution with reduced-dose DOACs (10, 15).

Notably, the discrepancy between complete resolution of the mobile mass on TTE at two weeks and possible minimal residual mural thrombus on CT at six weeks likely reflects the differing sensitivities of these imaging modalities. This observation underscores the complementary roles of TTE and CT in monitoring intracardiac thrombi and suggests that serial multimodality imaging may be warranted for complete resolution confirmation.

### Clinical implications and lessons learned

This case raises several important clinical considerations. First, LAAO does not confer comprehensive protection against all intracardiac thrombi. Up to 10% of intracardiac thrombi in nonvalvular AF originate from sites other than the LAA, indicating that LAAO provides incomplete protection against thromboembolism (14). Patients undergoing LAAO, particularly those with additional risk factors such as heart failure, enlarged cardiac chambers, and reduced ventricular function, may remain at significant risk for RAA thrombus formation.

Second, the indication for LAAO should be carefully scrutinized. In our patient, the CHA_2_DS_2_-VA score was 3, and the decision for LAAO was driven primarily by the contraindication to anticoagulation due to prior intracerebral hemorrhage. However, when LAAO is performed, patients are often transitioned off anticoagulation entirely, which may paradoxically leave other cardiac chambers unprotected against thrombus formation. Strict adherence to appropriate indications for LAAO is essential.

Third, this case supports targeted evaluation of the RAA during echocardiographic assessments in AF patients, particularly those with heart failure, biatrial enlargement, and prior LAAO. Incorporating RAA assessment into imaging protocols for these high-risk patients could facilitate earlier detection and treatment.

Fourth, the development of RAA thrombosis in our patient likely reflects generalized atrial disease and a persistent hypercoagulable milieu associated with atrial cardiomyopathy, rather than simply the withdrawal of anticoagulation. This distinction is clinically important because it suggests that some patients may require long-term antithrombotic therapy even after successful LAAO, particularly those with advanced structural heart disease.

## Conclusions

This case demonstrates that LAAO does not eliminate the risk of RAA thrombosis in patients with AF. The development of RAA thrombus following LAAO is likely multifactorial, involving the discontinuation of anticoagulation in the setting of persistent AF, heart failure, atrial cardiomyopathy, and atrial enlargement. Clinicians should maintain a high index of suspicion for RAA thrombosis in patients who have undergone LAAO, particularly those with additional risk factors. Targeted evaluation of the RAA during echocardiographic assessment should be considered in high-risk AF patients, particularly those with heart failure, biatrial enlargement, and prior LAAO. Careful individualization of anticoagulation strategies post-LAAO and strict adherence to appropriate LAAO indications are recommended. Anticoagulation with DOACs appears effective for resolving RAA thrombi, even at reduced doses. Further studies are warranted to determine the incidence of RAA thrombosis after LAAO and to establish optimal surveillance and management protocols for this clinically relevant finding.

## Data Availability

The original contributions presented in the study are included in the article/Supplementary Material, further inquiries can be directed to the corresponding author.

## Supplementary material

Supplementary Video S1 Echocardiography demonstrates a large mobile mass in the right atrium, arising from the region of the right atrial appendage.
